# Information, involvement, or partnership? Exploring intrapartum decision-making during the implementation of a team-based intervention to support woman-centred care

**DOI:** 10.1371/journal.pone.0358750

**Published:** 2026-09-23

**Authors:** Karin Johnson, Monica E. Nyström, Anna Ivert, Olof Stephansson, Amber Weiseth, Sissel Saltvedt, Malin Edqvist

**Affiliations:** 1 Division of Clinical Epidemiology, Department of Medicine Solna, Karolinska Institutet, Stockholm, Sweden; 2 Department of Women’s Health and Allied Health Professionals, Karolinska University Hospital, Stockholm, Sweden; 3 Department of Learning, Informatics, Management, and Ethics, Karolinska Institutet, Stockholm, Sweden; 4 Ariadne Labs, Harvard T.H. Chan School of Public Health, Boston, Massachusetts, United States of America; 5 Department of Women’s and Children’s Health, Department of Medicine Solna, Karolinska Institutet, Stockholm, Sweden; Deakin University Faculty of Health, AUSTRALIA

## Abstract

**Introduction:**

Despite being recognised as a critical component of high-quality care, shared decision-making is still not consistently achieved during childbirth. TeamBirth is a care process developed to promote intrapartum teamwork and shared decision-making, adapted to the Swedish context and implemented in two labour wards. This study aims to explore how care providers engage women and their partners in intrapartum decision-making during the implementation of the adapted Swedish version of TeamBirth.

**Methods:**

A qualitative design, based on 23 observations comprising 79 huddles, and 65 interviews with the care providers involved, was used. Data were collected April 2022 – June 2023, and analysed deductively using directed content analysis, guided by the Conceptual Framework for Patient and Family Engagement in Healthcare.

**Results:**

Four levels of engagement were found. *No Engagement* involved care providers giving instructions without explanations. *Providing Information* entailed brief updates followed by instructions, with limited opportunities for questions. *Involvement*, the most common level, included discussions and planning with women and their partners, although final decisions were made by care providers. *Partnership and Shared Leadership,* the least observed level, was characterised by dialogue and joint decisions. Barriers for engagement included limited Swedish proficiency, high workload, and critical situations, whereas continuous support, effective pain management, and use of the TeamBirth care process were enabling factors.

**Conclusions:**

Engagement in decision-making occurred on a continuum, with modifiable factors including workload, continuous support, and use of TeamBirth. Although conducted during the implementation of a care process designed to improve engagement, *Partnership and Shared Leadership* remained the least observed level.

## Introduction

Over the past decade, the importance of providing woman-centred and respectful care during childbirth has been increasingly recognised [1]. Respectful intrapartum care is defined as care that provides women with equitable access to evidence-based services that are safe, dignified, and responsive to individual needs and preferences [2]. It also encompasses informed decision-making, continuity of care, and supportive environments [3]. Within intrapartum care, shared decision-making (SDM) is strongly associated with positive birth experiences; however, many women report limited opportunities to participate in decisions regarding their care [4–6]. In Sweden, this is exemplified by data from the National Swedish Pregnancy Survey, which indicates that only 53% of women reported being involved in decision-making to the extent they desired [7].

“Nothing about me without me” has been used to summarise what SDM entails [8]. In intrapartum care, SDM is a dynamic process in which women and care providers collaborate to make decisions, guided by the woman’s preferences [9]. As greater attention is being directed toward SDM and toward women becoming active participants, this has, in turn, led to the use of a variety of concepts with slightly different but overlapping meanings, such as empowerment, engagement, enablement, participation, involvement, and activation [10]. While involvement, participation, activation, and empowerment often focus on the person receiving care [10], engagement is a broader concept that encompasses both relational and interactive dimensions [9]. Put differently, engagement may both foster and result from activation, empowerment, participation, and involvement, leading to SDM [9]. Engagement has been defined as the active collaboration of patients, their representatives, and health professionals across multiple levels of the healthcare system, including direct care, organisational design and governance, and policy making, with the aim of improving health and outcomes [11]. Engagement has further been widely recognised as a critical component of high-quality care, contributing to more responsive services [12,13], better patient experiences, and improved outcomes [14].

Carman and colleagues developed a conceptual framework for engagement in health care [11]. The framework outlines the forms that engagement can take on a continuum, from consultation, via involvement, to partnership and shared leadership. At the lower end of the continuum, those receiving care are involved but have limited power or decision-making authority, and care providers define the agenda. At the higher end, engagement is characterised by shared power and responsibility, with patients being active partners in planning and decision-making [11]. Furthermore, the framework outlines factors affecting engagement. Factors at the individual level include beliefs about and motivation to participate, health literacy, and education, while factors at the organisational level include policies, practices, and workplace culture. At the societal level, social norms, public policies and regulations affect providers [11].

Except for midwifery continuity of care (MCOC) [15,16], there is a notable lack of care models or interventions that effectively support care providers in engaging women and their partners in decision-making during childbirth. In Sweden, most women give birth in hospitals. Midwives are the primary care providers, supported by nurse assistants, while physicians are medically responsible for women with chronic conditions, high-risk pregnancies, childbirth complications, or operative births. Only a minority of women have access to continuity of care since MCOC in Sweden is largely directed towards vulnerable populations [17]. In the last decade, the TeamBirth care process was developed in the United States (US) to aid care providers in improving team communication and providing respectful intrapartum care [18,19]. A central aspect of this team-based care process is placing the woman at the centre of care and as an active participant in decision-making. Discussions, planning, and decision-making take place in the birthing room with the woman, her partner, and the care team. These interactions occur in brief meetings, called “huddles,” during which the woman’s preferences, labour progress, care plans, decisions, and the scheduled time of the next huddle are documented on a planning board visible to all participants. According to the process, anyone in the care team, as well as the woman or her partner, can initiate a huddle [18]. TeamBirth has been evaluated in the US context, where it has been associated with increased autonomy for women regarding decision-making [20,21].

This study is part of a larger project that aimed to examine how the TeamBirth care process was adapted to the Swedish context and implemented at a large obstetric unit comprising two labour wards. The project further aimed to explore how teamwork, patient safety, and engagement in decision-making were enacted during the implementation. The present study aimed to explore how care providers engaged women and their partners in intrapartum decision-making during the implementation of the adapted Swedish version of the TeamBirth care process.

## Methods

This study employed a qualitative research design using data collected through observations and interviews to explore the phenomenon of interest. Observations were used to obtain an in-depth understanding of interactions and behaviours within their natural context, allowing the study to capture the situational and environmental factors that shape participants’ actions and perspectives [22]. The interviews were semi-structured and conducted after each observation with all care providers who had participated in the huddles and were used to triangulate data and validate the findings [23,24].

### Study setting and participants

The study was conducted at two labour wards in a large teaching hospital in Sweden, geographically separated but organised under a single obstetric unit. Annual birth rates were approximately 4300 at site A and 3500 at site B. The two labour wards serve women with severe medical or psychiatric conditions, alongside those with low-risk pregnancies. A considerable population of women at both wards are immigrants and/or come from socially disadvantaged backgrounds. Care at the two labour wards is organised with a senior midwife at each shift who provides clinical guidance and support to less experienced colleagues. Medical decisions are predominantly provided by residents in training under the supervision of obstetricians. Midwives and nurse assistants work either day or night shifts, whereas physicians rotate across all hours. The care is medicalised, with high rates of interventions such as induction of labour, epidural analgesia, and oxytocin augmentation [25]. Time-outs are part of clinical practice when labour does not progress or in acute situations. During a time-out, the care team gathers with the woman and her partner in the birthing room to assess the progress of labour and jointly plan the next steps in care. The time-outs are inspired by the WHO Surgical Safety Checklist [26].

Eligible participants were women over 18 years of age who were in early labour or had sufficient pain relief to comprehend the study information and provide consent, and for whom either the woman or her partner had adequate proficiency in Swedish or English to understand the written and/or oral information. Care providers were eligible if they cared for a woman who had agreed to participate in the study.

### Observations and interviews

The observations were carried out by two of the researchers (KJ and AI) using an *“observer as participant”* approach [27], and they subsequently conducted the interviews. KJ and AI, a midwife and a resident, interacted to some extent with care providers, women and their partners during the observations. For example, they responded to questions but did not take part in huddles or other care activities. Since one of the project's aims was to study the implementation of the care process, data were collected during three periods: April to May 2022, September 2022, and June 2023. In total, 23 observations were conducted, 17 during day shifts, four during evening shifts, and two during night shifts. 12 took place at site A and 11 at site B. The 23 observations included 79 huddles, with the number of huddles per observation ranging from two to six. The duration of the huddles ranged from five to 80 minutes, with a median duration of 15 minutes. Further details regarding the observations are presented in S1 Table and S2 Table.

Each observation, except for one that involved only a midwife, followed a midwife–nurse assistant pair during their shift, aiming to attend all huddles they participated in. The observations were semi-structured and guided by the study aim, with a focus on communication and interactions between women, their partners, and care providers during huddles. No predefined checklist or categories were used. The huddles were initiated for various reasons. Some were clearly defined; for example, shift handovers, time-outs due to prolonged labour, or suspected foetal distress. Others were more informal, related to assessing labour progress, encouraging upright positions, or supporting pain management. Detailed notes were taken to document communication between care providers, women, and partners, and to record as much of the clinical and interpersonal dynamics as possible. Between huddles, the researchers stayed in the staff area to reflect and finalise notes. The two researchers did pilot observations and interviews at the start of the project. To ensure consistency, observation protocols were used in this phase, reminding the researchers of the aim and focus of the project. After each pilot observation, ME reviewed the notes from the observations and the interviews and provided feedback to improve their detail and clarity.

The pilot observations and interviews were considered to be of sufficient quality and were included in the analysis.

The interviews conducted with the care providers participating in the huddles were semi-structured using an interview guide to capture the focus areas of the entire research project (S3 Appendix). They were audio-recorded and subsequently transcribed verbatim.

Of the 79 potential participants in the huddles, 68 were interviewed: 26 midwives, 21 nurse assistants, eight midwifery students, five senior midwives, and eight obstetricians or residents. The interviews ranged from three to 30 minutes, with a median of 11. In the shorter interviews, the respondents did not answer all the questions; however, all participants responded to the first question, which concerned the workload during their shift. Eight participants were not interviewed because their shifts had ended, or they had become occupied with other duties.

### Ethics

All participants received written and verbal information about the study’s aim, the nature of participation, and their right to withdraw consent at any time. Recruiting women in labour for a study involving an additional observer was considered sensitive. Therefore, the eligibility criteria stipulated that women be either in early labour or have adequate pain relief. After receiving study information, women and their partners were provided private time to discuss participation and reach an informed decision. It was emphasised that declining participation would not affect the care they received.

Care providers also provided informed consent. If a care provider was assigned to a woman who consented to participate but preferred not to take part, the woman was offered the option to withdraw; however, this situation did not arise. The study is part of a larger research project, approved by the Swedish Ethical Review Authority (no 2020−02500, on October 10, 2020), with an amendment for the specific study (no 2021-06055-02, approved on December 6, 2021).

### Data analysis

**Fig 1:**
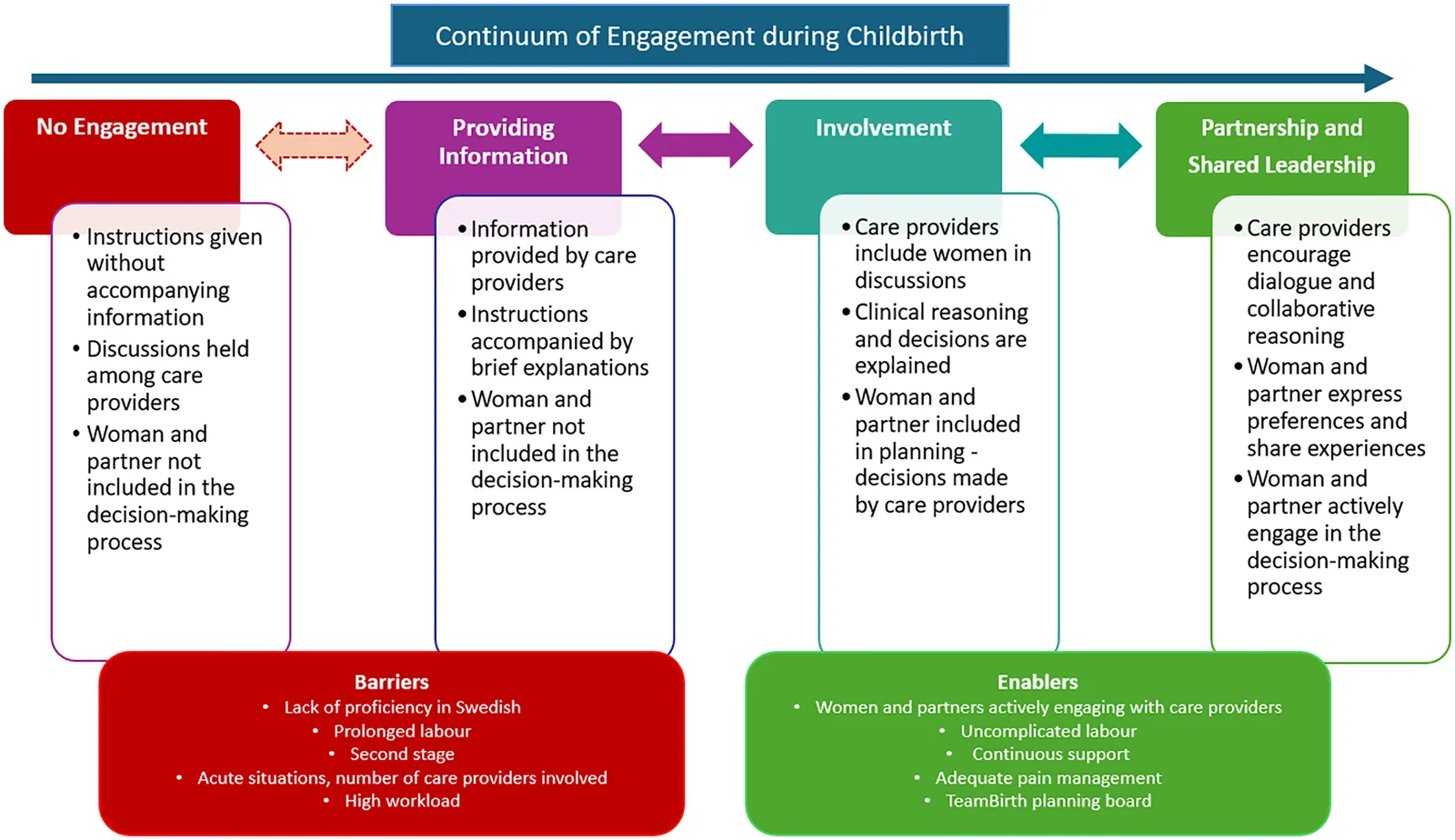
The Conceptual Framework for Engagement, adapted and applied to intrapartum care in the study. Illustrates and briefly describes the different types of engagement observed during huddles in the birthing room.

The analysis was carried out in several steps. All fieldnotes from the huddles were read repeatedly to ensure familiarity with the data. KJ then conducted the coding, followed by an iterative, collaborative process with ME and MEN. The observations were analysed using directed content analysis with a deductive approach [28], guided by the Conceptual Framework for Engagement [11]. Emerging findings were continuously discussed throughout this phase. The analytic process was iterative, involving movement between the whole (the observations) and the parts (meaning units and categories) across multiple cycles, allowing emerging insights to be revisited and reflected upon. Finally, the interviews were reviewed to triangulate data and deepen the understanding of the findings [29]. Analysis was limited to interview content in which care providers reflected on engaging women and partners in decision-making and factors influencing engagement. After KJ, ME, and MEN developed the preliminary findings, the other authors were invited to review and contribute their perspectives, which informed the final version of the analysis. During the analysis, the Conceptual Framework for Engagement [11] was slightly adapted to fit the context of intrapartum care within this study. For example, situations were identified where no engagement according to the framework could be observed, so this information was added, and the level labelled *Consultation* did not fully correspond to interactions in the birthing room and was therefore relabelled *Providing Information*. Factors influencing engagement were further categorised into barriers and enablers. The adaptations of the original framework are presented in Fig 1.

### Researcher characteristics and reflexivity

This study was conducted by a multidisciplinary research team, strengthening the trustworthiness and credibility of the findings. KJ and ME, both experienced midwives, contributed clinical insights from a midwifery perspective. AI, a resident, together with OS and SS, senior obstetricians, added complementary medical expertise. MEN and AW contributed external perspectives; MEN as an experienced implementation science researcher, and AW through expertise in designing and evaluating the TeamBirth care process in the US, also providing an international perspective. Prior to data collection, the researchers discussed and reflected on their individual preunderstandings of childbirth and decision-making. During data collection and analysis, reflexive notes and regular team discussions were used to critically examine emerging interpretations and the potential influence of the researchers’ professional backgrounds and perspectives. During the analysis in particular, interpretations were continuously questioned and discussed to obtain a deeper and more nuanced understanding of the data. The diversity of professional backgrounds further supported this ongoing reflexive process.

## Results

The levels of engagement, adapted to suit intrapartum care within the study context, are shown in Fig 1, along with factors influencing engagement.

The results are presented according to the adapted levels of engagement (Fig 1), and to three types of care measures identified as areas where women were, or were not, actively engaged during childbirth: *Care actions*, *Clinical procedures*, and *Medical interventions*. *Care actions* describe supportive and relational activities aimed at promoting women’s feelings of safety, comfort, and emotional well-being. These include offering food and fluids, encouraging mobility and changes in position, and providing or supporting pain relief strategies and pain management. *Clinical procedures* represent routine technical or monitoring activities conducted as part of standard intrapartum care at a labour ward, such as vaginal examinations, blood sampling, cardiotocography (CTG) monitoring, administration of medication, and intermittent catheterisation. *Medical interventions* refer to procedures initiated based on medical indications that may significantly influence the birth process or outcomes. They are performed to manage, assess, or accelerate the birth process, or to resolve complications during childbirth, and include amniotomy, augmentation of labour, foetal scalp lactate sampling, episiotomy, and operative birth. Table 1 presents all levels of engagement across the three types of care measures, with examples drawn from observations.

**Table 1:** Levels of engagement across the three types of care measures, with clinical examples from observations.

Levels of engagement	Care measures	Example from observations
**No engagement**	Care actions	*First-time mother, limited proficiency in Swedish. The midwife approaches the woman: “We’re going to give the handover to the incoming team here, bedside, and make a plan.”* *The midwifery student helps the woman lie on her side, with assistance from her husband.* *The student then goes to the computer to give the report; the midwife and the nurse assistant stand close together. Everyone speaks in low voices, making it difficult for the woman or her partner to hear what is being said.” (Observation 15, Site B)*
	Clinical procedures	*Second child, native Swedish. The woman is actively pushing in the lithotomy position, with two midwives, a nurse assistant, and two physicians present in the room. The birth may need to be completed with the help of vacuum extraction.* *Physician to the woman: “Do you find this very painful?”* *Woman: “No.”* *Midwife to physician: “Is she going to give birth without a pudendal block (PDB)?”* *The two physicians and the midwife discuss uterine inertia and note that the woman has a well-functioning epidural. The healthcare providers decide to postpone administering a PDB without consulting or informing the woman about their decision. (Observation 8, Site B)*
	Medical interventions	*Second child, native Swedish. There is a high workload in the delivery ward, which is why the midwife has left the delivery room to consult a physician. The midwifery student and the assistant nurse remain in the room with the woman and her partner.* *The midwife returns to the room, walks straight to the infusion pump, and turns off the oxytocin infusion without saying anything.* *Midwifery student to the responsible midwife: “You know what, we also need antibiotics.”* *Midwife: “Good, could you go and take care of that?”(Observation 8, Site B)*
**Providing information**	Care actions	*First-time mother, native Swedish. The woman is in the second stage of labour and is about to start pushing. A senior midwife has just entered the room to provide support; the responsible midwife and the nurse assistant are also present.* *Woman: “Is anything happening?”* *Midwife: “A lot is happening — the baby has moved further down than before. I’m thinking like this: the baby is doing well, and you’re very tired, but we’re not in a hurry.”* *The woman starts to cry.* *Midwife: “I feel so mean for saying that.”* *The midwife presses on the woman’s hips, then moves up towards her head: “I’m thinking we’ll sit on the birthing stool for a while, and that Carlos will sit behind you.” (Observation 17, Site A)*
	Clinical procedures	*First-time mother, native Swedish. The responsible midwife and the nurse assistant are present in the room. The midwife updates the TeamBirth board while speaking with the woman and her partner:* *“And the next vaginal examination will be at 19:20. You’re in the active phase now, and we examine every two hours unless something else happens.”* *The midwife marks an X on the TeamBirth board under “active phase” and again explains the descent phase and the pushing phase. (Observation 13, Site B)*
	Medical interventions	*First-time mother, native Swedish. A bedside time-out is planned, as the woman has been fully dilated for quite some time. The responsible midwife, assistant nurse, and senior midwife are present in the room.* *Senior midwife: “We’re going to gather our little team. Well, little—we’re a small group who’s here to take care of you. So now one of our physicians will come in.”* *The physician kneels down, looks the couple in the eyes, and says:* *“You’re probably wondering what I’m doing here. I’m here because, ultimately, this has been going on for a while, and you’ve been fully dilated for quite some time now. So, I’m here to see if I might need to help in some way — and in that case, the vacuum extractor is my strongest weapon.”* *The physician then turns to the senior midwife: “I’d like to examine her in the lithotomy position with some directed pushing. How long have you been sitting on the stool?”* *Midwife: “She just sat down.” (Observation 22, Site A)*
**Involvement**	Care actions	*First-time mother, native Swedish. The woman is experiencing increasingly painful contractions and has recently started using nitrous oxide for pain relief. The responsible midwife and the nurse assistant are present in the room.* *Midwife to the woman: “Breathe the gas a little longer — don’t take it away too early.”* *Woman: “My back, oh this back…”* *The nurse assistant and the midwife move closer to the bed.* *Midwife: “Maybe you should change position? Lie a bit on your side?”* *Woman: “Yes.”* *The midwife listens to the fetal heart rate, and the nurse assistant prepares for a position change by lowering the bed.* *Midwife to the woman: “Would you like a hot-pack?”* *Woman: “Yes.” (Observation 6, Site B)*
	Clinical procedures	*First-time mother, native Swedish. There has recently been a shift change. The responsible midwife, the midwifery student, and the nurse assistant are present in the room, getting acquainted with the couple.* *Midwife to the woman: “…We’d like to examine you, if that’s alright with you?”* *Woman: “Yes.”* *Midwife: “Just to see if the membrane is still intact. We’ll start by examining you and take it from there. And have you eaten?”* *Woman: “Yes, a little. But I’m so afraid of vomiting.” (Observation 11, Site B)*
	Medical interventions	*First-time mother, limited proficiency in Swedish. The woman has been fully dilated for one hour. A senior midwife has just entered the room to make a new assessment together with the responsible midwife and the nurse assistant.* *Midwife: “Now we need to plan ahead, because we want the baby to move further down. What we usually recommend, since we need to help the baby descend and the uterus needs a bit more strength, is to start an infusion that helps the uterus contract more strongly.”* *Woman: “Ah, okay.”* *Midwife: “Does that feel alright to you?”* *Woman: “Mm, okay.”* *Midwife: “If things progress quickly, we can turn it off.” (Observation 12, site A)*
**Partnership and shared leadership**	Care actions	*Second child, native Swedish. There has recently been a shift change. The responsible midwife and the midwifery student are present in the room, systematically going through the TeamBirth board and filling it in together with the woman and her partner.* *Midwife: “We’ll also be providing continuous support for you today. We’re a whole team who can help each other — me, the nurse assistant, and the student.”* *Woman and partner: “That sounds great.”* *Midwife: “And then we have this long arrow here (points to the bottom of the board) showing the different phases of labour. Right now, you’re about here (marks with an X under active phase), in the active part of labour. Then comes the descent phase, which people often forget — that even after you’re fully dilated, it can take some time for the baby to move down before it’s time to start pushing.”* *The woman and her partner confirm that they understand.* *The midwife sums up, emphasising that the board is dynamic and will be updated as labour progresses.* *The woman expresses a wish that her partner repeat what is being said if she’s using the nitrous oxide, since she had trouble hearing during her previous birth. The midwife adds this under “preferences” on the board. (Observation 5, site A)*
	Clinical procedures	*Third child, native Swedish. The woman is expecting her third child and is in the first stage of labour. The responsible midwife and the nurse assistant are present in the room.* *The woman says that she feels the urge to go to the toilet to urinate, but also feels some pressure downward during contractions. She says that she would like the midwife to examine her now instead of at 10 o’clock, as she wants to know “where she’s at” in the process.* *The midwife explains that she can help the woman urinate using a catheter instead, if getting up to the toilet feels too difficult, but the woman says she wants to try going to the toilet herself. The midwife agrees and says: “Go to the toilet, and we’ll examine you afterwards.” (Observation 2, site A)*
	Medical interventions	*Second child, limited proficiency in Swedish. The woman is expecting her second child, and the labour has not progressed for many hours. The epidural anaesthesia is not working properly, and she is in pain and exhausted. A resident doctor and a specialist doctor are in the room together with the responsible midwife, the midwifery student, and the nurse assistant. A new assessment of the situation has been made.* *Resident, facing the woman: **“**So it’s the same, up to this point. How are you feeling now?* *Woman: “I’m in pain.”* *Resident: “We’ve discussed it and are thinking that we could turn off the drip so that you can get some sleep tonight. Then we’ll see again tomorrow if there’s been any progress, once you and the baby have had some rest. Sometimes the body can start up on its own when it gets a break. Tomorrow we’ll see how you’re feeling. How do you feel about that plan?”* *Woman: “I want to have a C-section.”* *Resident: “Yes, you want to have a caesarean section?!” (Observation 10, site B)*

### No Engagement

*No Engagement* meant that care providers did not involve women in planning or decision-making. This was observed in 12 of the 79 huddles; however, only one huddle consisted solely of *No Engagement,* while the remaining 11 occurred alongside other levels of engagement (Table 2 and S2 Table). Furthermore, nine of the 12 huddles included an information exchange between care providers, either between midwives or between midwives and physicians, during handovers between shifts, when the primary midwife consulted a senior colleague, or during time-outs.

**Table 2:** Overview of observations: number of huddles, birthing woman characteristics, provider roles, shifts, and engagement levels.

Observation and number of huddles	Information about the birthing woman	Stage of labour	Profession and work experience of care providers (years)	Work shift	Level of involvement
Obs 1/ 4 huddles	First-time mother, non-native Swedish speaker	1^st^ and 2^nd^ stage	MW (<1), NA (12), sMW (10), PH (3)	Day shift	No engagement & providing information
Obs 2/ 2 huddles	Third child, native Swedish speaker	1^st^ stage	MW (2), NA (<1)	Day shift	Involvement & partnership and shared leadership
Obs 3/ 2 huddles	Second child, non-native Swedish speaker	1^st^ stage	MW (2), NA (<1)	Day shift	Providing information & involvement
Obs 4/ 3 huddles	First-time mother, native Swedish speaker	Latent phase	MW (1), NA (<1)	Day shift	Providing information & involvement
Obs 5/ 5 huddles	Second child, native Swedish speaker	1^st^ and 2^nd^ stage	MW (11), NA (<1), MWs^a^ (30)	Day shift	Involvement & partnership and shared leadership
Obs 6/ 5 huddles	First-time mother, native Swedish speaker	Latent phase and 1^st^ stage	MW (2.5), MW (19), NA (<1), NA (<1), MWs^a^ (3)	Night shift	Involvement
Obs 7/ 3 huddles	First-time mother, native Swedish speaker	Latent phase and 1^st^ stage	MW (<1), NA (4)	Night shift	Involvement
Obs 8/ 4 huddles	Second child, native Swedish speaker	2^nd^ and 3^rd^ stage	MW (2), NA (21), MWs^a^ (8), PH (>10)	Day shift	No engagement, providing information & involvement
Obs 9/ 5 huddles	First-time mother, native Swedish speaker	1^st^ and 2^nd^ stage	MW (8), NA (<1), MWs^a^ (7)	Day shift	Providing information & involvement
Obs 10/ 2 huddles	Second child, non-native Swedish speaker	1^st^ stage	MW (2), NA (28), MWs^a^ (3), PH (2)	Evening shift	Involvement & partnership and shared leadership
Obs 11/ 4 huddles	First-time mother, native Swedish speaker	Latent phase, 1^st^ and 2^nd^ stage	MW (14), NA (10), MWs^a^ (4)	Evening shift	Involvement
Obs 12/ 6 huddles	First-time mother, non-native Swedish speaker	1^st^ and 2^nd^ stage	MW (4), NA (>20), sMW (16), PH (2)	Day shift	No engagement, providing information & involvement
Obs 13/ 3 huddles	First-time mother, native Swedish speaker	Latent phase, 1^st^ and 2^nd^ stage	MW (2), NA (3)	Evening shift	Providing information & involvement
Obs 14/ 5 huddles	First-time mother, native Swedish speaker	1^st^ stage	MW (2), NA (0), MWs^a^ (5)	Day shift	Providing information & involvement
Obs 15/ 3 huddles	First-time mother, non-native Swedish speaker	Latent phase and 2^nd^ stage	MW (2), NA (21), MWs^a^ (5), PH (<1), PH (7)	Day shift	No engagement, providing information, involvement & partnership and shared leadership
Obs 16/ 3 huddles	Third child, non-native Swedish speaker	2^nd^ stage	MW (16), NA (<1)	Day shift	Providing information & involvement
Obs 17/ 3 huddles	First-time mother, native Swedish speaker	2^nd^ stage	MW (1), MW (16), NA (<1), sMW (8)	Day shift	No engagement, providing information & involvement
Obs 18/ 3 huddles	First-time mother, native Swedish speaker	1^st^ and 2^nd^ stage	MW (12), MW (<1), NA (<1), PH (20)	Day shift	No engagement, providing information & involvement
Obs 19/ 2 huddles	Third child, native Swedish speaker	1^st^ and 2^nd^ stage	MW (1.5), sMW (11), NA (2)	Day shift	Involvement & partnership and shared leadership
Obs 20/ 3 huddles	First-time mother, native Swedish speaker	Latent phase and 1^st^ stage	MW (10), MW (3), NA (<1)	Day shift	Providing information & involvement
Obs 21/ 2 huddles	First-time mother, native Swedish speaker	1^st^ stage	MW (8)	Day shift	Involvement
Obs 22/ 5 huddles	First-time mother, native Swedish speaker	2^nd^ stage	MW (<1), NA (<1), sMW (16), PH (<1), PH (20)	Evening shift	No engagement, providing information & involvement
Obs 23/ 3 huddles	First-time mother, non-native Swedish speaker	1^st^ and 2^nd^ stage	MW (3), NA (8), MWs^a^ (4)	Day shift	No engagement, providing information & involvement

MW: midwife/ NA: nurse assistant/ MWs: midwifery student/ sMW: senior midwife/ PH: physician; ^a^Work experience as a nurse.

The following care professionals were interviewed multiple times: sMW (HSbm3KJo) - 3 times; sMW (HSbm6KJo/AI) - 2 times; (HSbm2KJo) - 2 times; MW (Solnabm4AI) - 2 times; NA (HSusk3KJo/AI) - 2 times; NA (HSusk3KJo/AI) - 5 times; MWs (SolnaAI) - 2 times.

*No Engagement* was characterised by care providers giving the woman instructions without offering relevant information or a clear explanation. Regarding care actions, this included instances where care providers instructed the woman to change position without informing her of the reason or asking her to agree. Similarly, for clinical procedures and medical interventions, *No Engagement* referred to situations in which routine blood sampling or an oxytocin infusion was initiated or discontinued without explanation. In these instances, midwives simply informed the women of the intended action and then proceeded. During time-outs in which *No Engagement* was observed, discussions occurred between care providers without including women or their partners. In these situations, women were referred to in the third person rather than addressed directly. At times, options and alternatives had been discussed outside the room before the huddle was initiated, and decisions were merely presented to the women and their partners. In instances with *No Engagement,* women were often silent and accepted the decisions and instructions; however, in one case, a woman asked that a position change be delayed until pain relief had taken effect, but her request was disregarded by the nurse assistant (Table 1).

### Providing Information

*Providing Information* was observed consistently in eight of the 79 huddles and partially in 34 huddles, alongside instances of *Involvement* or *No Engagement*. It was never observed together with *Partnership and Shared Leadership* (Table 2 and S2 Table). Consistent with *Consultation* in the framework for engagement [11], *Providing Information* was characterised by midwives or physicians delivering information without involving women or their partners in the decision-making process. For example, in relation to care actions, midwives provided updates, information, and instructions to the woman. Unlike situations categorised as *No Engagement*, these instructions were accompanied by brief explanations, such as why a change in position or pushing technique was considered necessary. At times, midwives framed their instructions as suggestions yet proceeded without waiting for the woman’s response. In instances where huddles included time-outs in which clinical procedures and medical interventions were proposed and carried out, information was given, but with limited time for questions, and the reasoning behind the decisions was not provided (Table 1). Women and their partners typically responded with brief acknowledgements, such as “yes”, “no”, or a nod.

### Involvement

*Involvement* was observed in the majority of huddles, being the only level of engagement in 31 of the 79 huddles, and present alongside other levels of engagement in an additional 34 huddles (Table 2 and S2 Table). This level was characterised by care providers involving women and their partners in planning and decision-making. Although midwives and physicians still made the decisions, communication often took the form of discussions in which the woman and her partner asked questions and expressed preferences.

In relation to care actions, midwives facilitated involvement by presenting options and seeking the woman’s input in discussions regarding pain relief management, position changes, or vaginal examinations. They occasionally invited women to express their preferences regarding the timing of the next assessment. At times, midwives also used alternative methods to convey information and enhance understanding, for example, demonstrating the baby’s descent using a doll and pelvic model. There were several observations of *Involvement* in relation to clinical procedures, but only a few regarding medical interventions, such as performing an amniotomy or augmenting labour with oxytocin. However, women and their partners were involved in discussions about mode of birth, primarily in relation to the potential need for caesarean section. This occurred in three cases of protracted labour when the baby showed no signs of distress. The degree to which midwives and physicians clearly explained their reasons for recommending certain assessments or interventions varied. Nevertheless, they frequently offered more suggestions and used less directive language, employing phrases such as *“It would be good if…”* or *“Maybe you could…”* (Table 1).

### Partnership and Shared Leadership

*Partnership and Shared Leadership* was the level least often observed, occurring consistently in only one huddle, and partially in four, together with *Involvement* (Table 2 and S2 Table). Here, women actively set the agenda and made decisions about their care together with the midwives. Instances characterised by *Partnership and Shared Leadership* displayed an ongoing dialogue between the birthing couple and the care providers, and most of them occurred with midwives (Table 2). Information was followed by responses from the woman and her partner, who in turn shared reflections, preferences, or experiences. Recommendations were accompanied by explanations and discussions of possible scenarios, including their pros and cons. Regarding care actions, midwives supported women by inviting them to express preferences, emphasising that their role was to provide support in accordance with the woman’s wishes (Table 1).

At times, women assumed a leading role, negotiating midwives’ suggestions, for example, when a woman asked the midwife to perform a vaginal examination earlier than planned. *Partnership and Shared Leadership* was also observed in five instances involving clinical procedures or medical interventions. In our observations, we found no situations in which this level of engagement led women to decline medical interventions suggested by care providers. Instead, women expressed specific preferences regarding the conduct of the intervention, for example, requesting a female physician to perform foetal lactate sampling. Similar to *Involvement*, midwives used phrases that signalled openness when presenting suggestions to the woman (e.g., *“I would recommend”, “I could”, “I believe that”)*, but went further by fostering engagement with statements such as *“Does that sound okay?”* or *“Because it is you who decides…”* (Table 1).

### Factors affecting engagement

In this study, both barriers and enablers to achieving engagement were identified. We observed that engagement was affected when women or their partners had limited proficiency in Swedish. In these instances, care providers used simpler language and provided fewer explanations. This was also expressed in the interviews, where midwives and nurse assistants described how low proficiency in Swedish, combined with partners acting as interpreters, complicated efforts to support engagement.

*“It was a bit harder today to get the woman and her partner involved, since they spoke a different language. He had to translate everything for her.” (Midwife, observation 16, Site A)*.

The course of labour also influenced the level of engagement. Prolonged labour and the intensity of contractions, for example, during the second stage, were barriers to higher levels of engagement. Huddles occurring during the second stage most often involved *No Engagement* or *Providing Information* (Table 2 and S2 Table). Midwives reported that promoting engagement became more challenging when labour intensified and women became increasingly focused.

*“It was difficult because she was having such intense contractions, and she didn’t have much pain relief either. She only had the gas, and she was very tired. I always find it difficult at the end to get a sense of how things are going.” (Midwife, observation 17, Site A)*

Barriers further included complications occurring during labour, for example, in instances where there were concerns about foetal well-being. These situations often led to more care providers participating in huddles. As the number of care providers and the seriousness of the situation increased, fewer instances of engaging women in decision-making were observed.

Factors enabling engagement included women having uncomplicated labours and adequate pain management. In addition, women and partners who took the initiative to engage with care providers facilitated higher levels of involvement. Midwives reported that when women or their partners actively communicated by asking questions and expressing their views and preferences, it was easier to engage them in decision-making.

*“The couple were really communicative too. They expressed their needs… and also the partner’s needs. So, it was easy to do things the way they wanted, and that leads to a really good communication, for them and us… because we feel confident about what they expect and what they want…” (Midwifery student, observation 11, Site B)*.

The ability to provide continuous support during labour further aided midwives in engaging and involving women. Both midwives and nurse assistants often mentioned workload as either a facilitator or a barrier to engagement. The TeamBirth planning board was described as a tool that predominantly enabled care providers to achieve higher levels of engagement. They particularly mentioned the designated spaces for documenting preferences, the plan, and the progress of labour and how this supported a shared mental model of the situation.

*“… I think it’s useful to get a more complete picture, and to make follow-ups easier. To understand what has happened before, what the plan was, and what the plan is now — so that everyone’s on the same page. Otherwise, it’s easy to lose information...” (Resident, observation 10, Site B).*

## Discussion

We found that the Conceptual Framework for Engagement (15) was useful for analysing involvement in decision-making during childbirth, highlighting engagement as a process occurring along a continuum rather than as a binary event. Previous studies on care providers’ attitudes towards engaging women in decision-making during childbirth often emphasise ethically challenging situations, such as the need to prioritise foetal wellbeing over maternal autonomy [30]. However, focusing primarily on such scenarios may obscure engagement as an everyday clinical process. Since integrating SDM into daily practice often requires behavioural change among care providers [31], this slightly adapted framework could offer a useful starting point for discussion and reflection.

In addition to tailoring the three original levels of engagement to reflect how engagement is manifested during intrapartum care, we identified an additional level of non-engagement, termed *No Engagement*. At this “zero-level”, care was non-participatory and directive, indicating an authoritarian and paternalistic approach. This aligns with Bohren’s typology of mistreatment, particularly the category describing poor rapport between women and providers [32]. In the present study, this was reflected in insufficient communication, women being treated as passive participants, dismissal of their wishes, and the resulting objectification [32].

It should be emphasised that situations labelled as *No Engagement* were predominantly observed in huddles together with *Providing Information*, and only one huddle consisted solely of *No Engagement*. *Providing Information* represents the lowest level of engagement on the continuum and may also be linked to disrespectful care [32], as the information provided by care providers was very brief and delivered with limited time before care actions, clinical procedures, or medical interventions were carried out. Engagement at the low end of the continuum occurred despite the study being conducted during the implementation of the TeamBirth care process, designed specifically to enhance communication and SDM processes [18,19], and despite care providers being aware that they were being observed. Frequently cited reasons for not engaging more actively with women included high workload and stress [33]. Another possible explanation is that the professions involved in intrapartum care may be unaware that certain behaviours, particularly more subtle forms, constitute disrespectful care [34]. However, it should be noted that information can be an important prerequisite for the ability to engage later in a process.

*Involvement* and *Partnership and Shared Leadership* reflected more favourable situations, characterised by predominantly enabling factors and fewer barriers. Factors highlighted in previous research as positively influencing SDM from the care provider perspective, but not explicitly observed or mentioned in our interviews, include trusting relationships among care providers, effective communication skills, and a supportive work culture [35,36]. Although good relationships were not directly discussed in the interviews, care providers sometimes reported the opposite, describing certain huddles as “messy” or “unstructured”, indicating that team collaboration was not always effective.

The highest level, but also the least frequently observed, was *Partnership and Shared Leadership*. Notably, in huddles where this level was present, women and their partners were more actively engaged in dialogue with midwives. These huddles involved the intended use of TeamBirth, including the planning board, designed to facilitate a shared understanding of the clinical situation and to support women’s participation in decision-making [18,19].

The barriers and enablers identified in this study are consistent with previous research [31,37]. The second stage of labour and less favourable situations, such as when complications arise, were both observed and highlighted by care providers as particularly challenging. Educational efforts, including the integration of involvement and SDM practices into training in obstetric emergencies, may represent a way forward to improve these situations. MCOC models have, in other contexts, been shown to contribute to engagement and decision-making [4,16]. However, as most Swedish women do not have access to relational care throughout pregnancy, labour, and the postpartum period, TeamBirth offer a means for care providers, birthing women, and their partners to engage in decision-making.

Finally, it is worth highlighting that most huddles included more than one level of engagement. In our observations, we did not identify any consistent patterns indicating that engagement consistently began at a low level and increased over time, or vice versa. Rather, this pattern points to the complexity of both clinical situations and the engagement process in intrapartum care, challenging assumptions of a gradual progression from information provision to more reciprocal forms of engagement. While such progression may occur in some situations, the analysis of barriers and enablers suggests that engagement is a multifactorial process [38–40], in which a single enabler, for example, a labouring woman and partner being engaged and motivated to participate, may counteract not only one but several barriers.

### Strengths and limitations

A key strength of this study is the combination of observations and interviews. Using a deductive approach guided by a theoretical framework allowed us to focus the analysis on theory related to engagement, thereby strengthening the interpretation of data in relation to existing knowledge [41]. Observations provide advantages over relying only on interview data since participants’ descriptions in the social context of an interview may differ substantially from their actual behaviours in the clinical setting [42]. Valuable insights can emerge from routine, everyday practices that participants may not consciously recognise and therefore do not report [22,43]. Furthermore, conducting pilot observations and interviews and receiving feedback from a senior researcher enhanced the quality and consistency of the data. Incorporating interview data to triangulate the findings further enhanced the study’s rigour and strengthened the credibility and trustworthiness of the results.

The limitations of the study should also be acknowledged. This is a sub-study within a larger research project examining the implementation of the Swedish version of the TeamBirth care process, and how SDM and patient safety behaviours were enacted. This required the observers to cover multiple areas during observations, which may have affected their focus. Pilot observations were therefore valuable in training the observers to capture situations and interactions, enabling analysis for all areas. The interview questions also related to all topics, which may have influenced the participants’ responses.

Having two researchers conduct the observations and interviews can be viewed as a limitation, since professional background may influence what is observed [44] and lead to less consistency in the data collected. Moreover, conducting observations in a familiar setting poses challenges related to pre-understanding. For example, certain forms of disrespectful care may be normalised among care providers [34], and consequently, such instances might not have been recorded. Additionally, since observations in the birthing room were only conducted during huddles, we are not aware of how the process in between huddles affected engagement in SDM. The transferability of the findings is therefore only relatable to similar contexts [45], i.e., team collaboration situations in this type of intrapartum care.

## Conclusion

Engagement in decision-making occurred along a continuum and was shaped by modifiable factors such as workload, continuous support, and the use of TeamBirth. Despite the study being conducted during the implementation of a process specifically designed to enhance engagement, the highest level, *Partnership and Shared Leadership*, was rarely observed. The conceptual framework visualises engagement as a continuum, providing a tool to support reflection, facilitate discussion, and promote behavioural change in clinical practice.

## Supporting information

S1 Table Overview of data collection by period and method. (DOCX)

S2 Table Overview of the reason for conducting a huddle, number of care providers, stage of labour, and levels of engagement for each huddle in the observations. (DOCX)

S3 Appendix Interview guide. (DOCX)

## Abbreviations

MCOC: Midwifery continuity of care
RMC: Respectful maternity care
SDM: Shared decision-making
US: United States
