# Supplementary material for: Information, involvement, or partnership? Exploring intrapartum decision-making during the implementation of a team-based intervention to support woman-centred care
Source: PLoS One. 2026 Sep 23;21(9):e0358750. doi: 10.1371/journal.pone.0358750 (PMC13600533; doi:10.1371/journal.pone.0358750)
Supplement: Supplementary file 1 [file pone.0358750.s001.docx]

S1 Table. Overview of data collection by period and method

|  | Observationsᵃ (N=23) | Huddles (N=79) | Interviewsᵇ (N=65) |
| --- | --- | --- | --- |
| First period  (April to May 2022) | 17 | 60 | 52 |
| Second period  (September 2022) | 4 | 13 | 9 |
| Third period  (June 2023) | 2 | 6 | 7 |
| Total | 23 | 79 | 68 |

ᵃObservations covered 17 day, 4 evening, and 2 night shifts / ᵇInterviews included 26 midwives, 5 senior midwives, 8 midwifery students, 18 nurse assistants, and 8 physicians
