# Supplementary material for: Information, involvement, or partnership? Exploring intrapartum decision-making during the implementation of a team-based intervention to support woman-centred care
Source: PLoS One. 2026 Sep 23;21(9):e0358750. doi: 10.1371/journal.pone.0358750 (PMC13600533; doi:10.1371/journal.pone.0358750)
Supplement: Supplementary file 2 [file pone.0358750.s002.docx]

S2 Table. Overview of the reason for conducting a huddle, number of care providers, stage of labour, and levels of engagement for each huddle in the observations

| **Observation** | **Huddle** | **Reason for**  **conducting huddle** | **Number of**  **care providers** | **Stage of**  **labour** | **Levels of engagementᵃ** | **No Engagementᵇ** | **Providing Informationᵇ** | **Involvementᵇ** | **Partnership & Shared Leadershipᵇ** |
| --- | --- | --- | --- | --- | --- | --- | --- | --- | --- |
| Obs 1 | 1 | Assess labour progress | 2 | First stage | 1, 1, 1 |  | x |  |  |
|  | 2 | Assess labour progress | 3 | Second stage | 1, 1 |  | x |  |  |
|  | 3 | Assess labour progress | 2 | Second stage | 1 |  | x |  |  |
|  | 4 | Time-out | 4 | Second stage | 1, 1, 0, 1, 1, 1 | x | x |  |  |
| Obs 2 | 1 | Assess labour progress | 2 | First stage | 3, 3, 3 |  |  |  | x |
|  | 2 | Assess labour progress | 2 | First stage | 3, 2 |  |  | x | x |
| Obs 3 | 1 | Suspected fetal distress | 2 | First stage | 1, 1, 2, 1, 2, 2, 1 |  | x | x |  |
|  | 2 | Assess labour progress | 2 | First stage | 2, 1, 1 |  | x | x |  |
| Obs 4 | 1 | Induction | 2 | Latent phase | 2, 2, 2 |  |  | x |  |
|  | 2 | Induction | 2 | Latent phase | 2 |  |  | x |  |
|  | 3 | Induction | 2 | Latent phase | 1, 2, 2, 2 |  | x | x |  |
| Obs 5 | 1 | Initial meeting | 3 | First stage | 3, 2, 3 |  |  | x | x |
|  | 2 | Assess labour progress | 3 | First stage | 2, 2, 2, 2, 2 |  |  | x |  |
|  | 3 | Urge to push | 3 | Second stage | 2, 2 |  |  | x |  |
|  | 4 | Assess labour progress | 3 | Second stage | 2, 2, 2, 2, 2, 2 |  |  | x |  |
|  | 5 | Pain relief | 3 | Second stage | 2, 2 |  |  | x |  |
| Obs 6 | 1 | Pain relief | 2 | Latent phase | 2, 2, 2 |  |  | x |  |
|  | 2 | Assess labour progress | 2 | First stage | 2, 2, 2, 2, 2 |  |  | x |  |
|  | 3 | Handover | 5 | First stage | 2 |  |  | x |  |
|  | 4 | Assess labour progress | 3 | First stage | 2, 2, 2, 2 |  |  | x |  |
|  | 5 | Assess labour progress | 3 | First stage | 2, 2, 2 |  |  | x |  |
| Obs 7 | 1 | Admission decision | 2 | Latent phase | 2, 2, 2 |  |  | x |  |
|  | 2 | Handover | 4 | Latent phase | 2 |  |  | x |  |
|  | 3 | Assess labour progress | 2 | First stage | 2, 2, 2 |  |  | x |  |
| Obs 8 | 1 | Assess labour progress | 2 | Second stage | 1, 2, 2, 2, 2, 2 |  | x | x |  |
|  | 2 | Assess labour progress | 3 | Second stage | 1, 2, 1, 2, 0, 0, 1, 1, 1, 1, 0, 1, 0, 1 | x | x | x |  |
|  | 3 | Directed pushing | 5 | Second stage | 1, 1, 1, 0, 1 | x | x |  |  |
|  | 4 | Handover | 5 | Third stage | 1 |  | x |  |  |
| Obs 9 | 1 | Assess labour progress | 3 | First stage | 2, 1, 2, 2, 2, 2, 2 |  | x | x |  |
|  | 2 | Assess labour progress | 3 | First stage | 2, 1, 2, 2, 2 |  | x | x |  |
|  | 3 | Assess labour progress | 3 | Second stage | 2, 1, 2, 2, 2 |  | x | x |  |
|  | 4 | Assess labour progress | 3 | Second stage | 2, 2, 2, 2, 2 |  |  | x |  |
|  | 5 | Handover | 4 | Second stage | 2 |  |  | x |  |
| Obs 10 | 1 | Pain relief | 5 | First stage | 2, 2, 2, 2, 2 |  |  | x |  |
|  | 2 | Time-out | 5 | First stage | 2, 2, 2, 2, 3, 2, 2 |  |  | x | x |
| Obs 11 | 1 | Initial meeting | 3 | Latent phase | 2, 2, 2, 2, 2, 2 |  |  | x |  |
|  | 2 | Assess labour progress | 3 | First stage | 2, 2, 2 |  |  | x |  |
|  | 3 | Assess labour progress | 3 | First stage | 2, 2, 2, 2 |  |  | x |  |
|  | 4 | Assess labour progress | 3 | Second stage | 2, 2 |  |  | x |  |
| Obs 12 | 1 | Initial meeting | 3 | First stage | 1, 2 |  | x | x |  |
|  | 2 | Assess labour progress | 2 | Second stage | 2, 2, 2, 1 |  | x | x |  |
|  | 3 | Assess labour progress | 3 | Second stage | 1, 2, 2 |  | x | x |  |
|  | 4 | Time-out | 4 | Second stage | 0, 1, 1, 0, 1, 1, 0 | x | x |  |  |
|  | 5 | Time-out | 5 | Second stage | 2, 1, 1, 2 |  | x | x |  |
|  | 6 | Directed pushing | 5 | Second stage | 0, 1, 1 | x | x |  |  |
| Obs 13 | 1 | Initial meeting | 2 | Latent phase | 2, 1 |  | x | x |  |
|  | 2 | Huddle at TB board | 2 | First stage | 2, 1 |  | x | x |  |
|  | 3 | Assess labour progress | 2 | Second stage | 1, 2 |  | x | x |  |
| Obs 14 | 1 | Assess labour progress | 3 | First stage | 2, 2, 2, 2 |  |  | x |  |
|  | 2 | Huddle at TB board | 3 | First stage | 2, 2 |  |  | x |  |
|  | 3 | Huddle at TB board | 3 | First stage | 2, 2 |  |  | x |  |
|  | 4 | Huddle at TB board | 3 | First stage | 2 |  |  | x |  |
|  | 5 | Handover | 5 | First stage | 1 |  | x |  |  |
| Obs 15 | 1 | Handover | 5 | Latent phase | 1, 2, 2, 2, 2 |  | x | x |  |
|  | 2 | Assess labour progress | 4 | Second stage | 2, 2, 2 |  |  | x |  |
|  | 3 | Fetal lactate + handover | 6 | Second stage | 2, 2, 2, 2, 2, 3, 2, 2, 2, 2, 0 | x |  | x | x |
| Obs 16 | 1 | Initial meeting | 2 | Second stage | 2, 1 |  | x | x |  |
|  | 2 | Suspected fetal distress | 2 | Second stage | 1, 1, 2, 1, 2 |  | x | x |  |
|  | 3 | Directed pushing | 3 | Second stage | 1, 1, 2 |  | x | x |  |
| Obs 17 | 1 | Pain relief | 2 | Second stage | 0 | x |  |  |  |
|  | 2 | Assess labour progress | 3 | Second stage | 1, 2, 2, 1, 1, 2, 1, 1, 0 | x | x | x |  |
|  | 3 | Directed pushing | 3 | Second stage | 1, 1, 1, 2, 1 |  | x | x |  |
| Obs 18 | 1 | Handover | 3 | First stage | 2, 2, 2 |  |  | x |  |
|  | 2 | Assess labour progress | 3 | Second stage | 1, 1, 1, 1 |  | x |  |  |
|  | 3 | Time-out | 5 | Second stage | 1, 2, 2, 1, 1, 0, 0 | x | x | x |  |
| Obs 19 | 1 | Assess labour progress | 2 | First stage | 2, 2, 3, 2, 2, 2, 2, 2 |  |  | x | x |
|  | 2 | Handover | 5 | Second stage | 2 |  |  | X |  |
| Obs 20 | 1 | Assess labour progress | 3 | Latent phase | 2, 2, 1, 1, 2, 2 |  | x | x |  |
|  | 2 | Assess labour progress | 3 | First stage | 2, 1, 2, 2, 2, 1 |  | x | x |  |
|  | 3 | Assess labour progress | 4 | First stage | 1, 2, 2, 2, 1 |  | x | x |  |
| Obs 21 | 1 | Assess labour progress | 1 | First stage | 2, 2, 2 |  |  | x |  |
|  | 2 | Assess labour progress | 1 | First stage | 2, 2, 2 |  |  | x |  |
| Obs 22 | 1 | Time-out | 3 | Second stage | 1, 0 | x | x |  |  |
|  | 2 | Suspected fetal distress | 3 | Second stage | 2, 1, 2 |  | x | x |  |
|  | 3 | Time-out | 4 | Second stage | 1, 2 |  | x | x |  |
|  | 4 | Time-out | 4 | Second stage | 1 |  | x |  |  |
|  | 5 | Time-out + Directed pushing | 5 | Second stage | 1, 1, 0, 2, 1 | x | x | x |  |
| Obs 23 | 1 | Assess labour progress | 3 | First stage | 2 |  |  | x |  |
|  | 2 | Assess labour progress | 3 | Second stage | 1, 1 |  | x |  |  |
|  | 3 | Handover | 4 | Second stage | 1, 0, 2, 1, 2, 1, 1 | x | x | x |  |

ᵃLevel 1: No engagement, Level 2: Providing information, Level 3: Involvement, Level 4: Partnership & shared leadership

ᵇX indicates the level of engagement observed during the observation and huddle
