# Supplementary material for: Information, involvement, or partnership? Exploring intrapartum decision-making during the implementation of a team-based intervention to support woman-centred care
Source: PLoS One. 2026 Sep 23;21(9):e0358750. doi: 10.1371/journal.pone.0358750 (PMC13600533; doi:10.1371/journal.pone.0358750)
Supplement: Supplementary file 3 [file pone.0358750.s003.docx]

**S3 Appendix. Interview guide**

The interview guide was developed to evaluate the implementation of TeamBirth, including participation/engagement and patient safety. For the purposes of this study, responses to questions under subheadings A and D were analysed in detail. However, all interviews were reviewed in full, and any content relevant to the study’s aim was included in the analysis.

A) **Overall questions**

- How has your work shift been today?

B) **Questions regarding the implementation of TeamBirth**

- How was it for you to use TeamBirth in your work today?
- How was it to use the planning board?
- Was there any part of the board that was especially good, or particularly difficult or unclear?
- How was it to do the huddles with the woman and her partner?
- How was it to do the huddles together with the other care providers (depending on who participated)?
- How do you think it affected the woman?
- How did it affect the partner?
- Was there anything that worked especially well?
- Was there anything that worked less well?

C) **Questions regarding patient safety**

- How do you think the collaboration worked today? (with the doctor, assistant nurse, the couple, etc.
- How do you think the communication worked today?
- Have the TeamBirth planning board and the huddles contributed in any way?
- What could have made the collaboration better today?
- What made collaboration more difficult today?
- Did you feel that the members of the team shared the same understanding of what was happening in the room?
- During this shift, do you think TeamBirth contributed to patient safety?
- Based on what we have talked about now, what thoughts do you have about patient safety during childbirth (teamwork, communication)? Anything that came to mind during this shift?

D) **Questions regarding participation/engagement**

- What does involvement and participation mean to you? How do you involve the couple?
- Was there anything related to involvement or participation that came to mind during this shift?

E) **Additional questions**

- Additional questions based on the observation. (This question was intended to remind the observers to ask questions if anything came up during the huddles.)

Is there anything else you would like to add?
