# Supplementary material for: Information, involvement, or partnership? Exploring intrapartum decision-making during the implementation of a team-based intervention to support woman-centred care
Source: PLoS One. 2026 Sep 23;21(9):e0358750. doi: 10.1371/journal.pone.0358750 (PMC13600533; doi:10.1371/journal.pone.0358750)
Supplement: Supplementary file 4 [file pone.0358750.s005.docx]

# Response to Reviewers and Changes to meet Journal Requirements

Dear Editor and Reviewers,

Thank you for your thoughtful and constructive comments, which have helped improve the manuscript. We have addressed each comment point by point below, and all changes made to the manuscript are highlighted in red.

**Reviewer #1:** This is a well written manuscript on an important topic that is highly relevant to current global focus on respectful care and informed/shared decision-making during labour and birth. The research process has been managed with rigour and integrity. I have only one minor comment:

On page 9, the section headed '2.5 Researcher characteristics and reflexivity' describes the researcher characteristics very clearly but does not contain any reflexivity. Please include some examples of reflexivity from authors, to justify your statement: 'The diversity of professional backgrounds fostered continuous reflexivity, supported by discussions throughout the study'.
I look forward to seeing this and other manuscripts emerging from this study being published.

**Author’s response:** Thank you for your kind and positive feedback regarding our manuscript. We agree that the previous description did not provide sufficient examples of how reflexivity was practiced throughout the study. We have therefore revised the section “Researcher characteristics and reflexivity” to include specific examples of our reflexive approach **(p. 8, lines 227-234):**

Prior to data collection, the researchers discussed and reflected on their individual preunderstandings of childbirth and decision-making. During data collection and analysis, reflexive notes and regular team discussions were used to critically examine emerging interpretations and the potential influence of the researchers’ professional backgrounds and perspectives. During the analysis in particular, interpretations were continuously questioned and discussed to obtain a deeper and more nuanced understanding of the data. The diversity of professional backgrounds further supported this ongoing reflexive process.

**Reviewer #2:** This is a very well written paper with a comprehensive methodological approach to explore shared-decision making in early labour care. The study provides important insights about effective shared-decision making approaches and barriers to practice. I have included some very minor revisions to address below.

**Author’s response:** Thank you for your encouraging words and for highlighting the relevance of our methodological approach and findings. We appreciate your thoughtful comments and have addressed the suggested revisions below.

**Introduction**
Line 124-125- Increased autonomy in decision making of whom?

**Author’s response:** Thank you for pointing this out. We have clarified that the statement refers to women’s autonomy in decision-making. The sentence has been revised as follows **(p. 4, lines 107-108):**

TeamBirth has been evaluated in the US context, where it has been associated with increased autonomy for women regarding decision-making [20, 21].

**Study setting and participants**
Line 152- what are timeouts?

**Author’s response:** Thank you for highlighting the need to clarify this term. We have now added a brief description of time-outs in the manuscript **(p. 5, lines 135-139):**

Time-outs are part of clinical practice when labour does not progress or in acute situations. During a time-out, the care team gathers with the woman and her partner in the birthing room to assess the progress of labour and jointly plan the next steps in care. The time-outs are inspired by the WHO Surgical Safety Checklist [26].

**Observations and interviews**
Were the observations structured/semi-structured/unstructured, i.e., did you have a form of aspects of care you were looking for. Or was as much detail about what occurred documented (i.e., unstructured).

**Author’s response:** Thank you for raising this point. The observations were semi-structured and guided by the study aim, with a focus on communication and interactions between care providers, women, and partners during huddles. No predefined checklist or categories were used; rather, detailed field notes were taken to capture as much as possible of the clinical and interpersonal dynamics occurring during the huddles. This has now been clarified in the Methods section **(p. 6, lines 159-162):**

The observations were semi-structured and guided by the study aim, with a focus on communication and interactions between women, their partners, and care providers during huddles. No predefined checklist or categories were used.

**Results**
Line 256- CTG in full before acronym

**Author’s response:** Thank you for pointing this out. CTG has now been written out in full at first mention, as follows **(p. 9, lines 247-250):**

*Clinical procedures* represent routine technical or monitoring activities conducted as part of standard intrapartum care at a labour ward, such as vaginal examinations, blood sampling, cardiotocography (CTG) monitoring, administration of medication, and intermittent catheterisation.

# **Additional changes to meet Journal Requirements:**

1. When submitting your revision, we need you to address these additional requirements.

Please ensure that your manuscript meets PLOS ONE's style requirements, including those for file naming. The PLOS ONE style templates can be found at

<https://journals.plos.org/plosone/s/file?id=wjVg/PLOSOne_formatting_sample_main_body.pdf> and

<https://journals.plos.org/plosone/s/file?id=ba62/PLOSOne_formatting_sample_title_authors_affiliations.pdf>

**Author’s response:** Thank you for highlighting this. The manuscript has been carefully checked and revised to ensure compliance with the PLOS ONE style and formatting requirements, including the file naming requirements.

2. We note that the grant information you provided in the ‘Funding Information’ and ‘Financial Disclosure’ sections do not match.

When you resubmit, please ensure that you provide the correct grant numbers for the awards you received for your study in the ‘Funding Information’ section.

**Author’s response:** Thank you for pointing this out. The corrected grant numbers have now been provided in the Funding Information section.

3. Thank you for stating the following financial disclosure:

The study was supported by funding from the Swedish Research Council for Health, Working Life and Welfare (Grant no. 2020-01209) and from Region Stockholm (Grant no. FoUI-974668).

Please state what role the funders took in the study.  If the funders had no role, please state: "The funders had no role in study design, data collection and analysis, decision to publish, or preparation of the manuscript." If this statement is not correct you must amend it as needed.

Please include this amended Role of Funder statement in your cover letter; we will change the online submission form on your behalf.

**Author’s response:** Thank you for this comment. As the funders had no role, the following line has been included in the revised cover letter: The funders had no role in study design, data collection and analysis, decision to publish, or preparation of the manuscript.

4. We note that you have indicated that there are restrictions to data sharing for this study. For studies involving human research participant data or other sensitive data, we encourage authors to share de-identified or anonymized data. However, when data cannot be publicly shared for ethical reasons, we allow authors to make their data sets available upon request. For information on unacceptable data access restrictions, please see <http://journals.plos.org/plosone/s/data-availability#loc-unacceptable-data-access-restrictions>.

Before we proceed with your manuscript, please address the following prompts:

a) If there are ethical or legal restrictions on sharing a de-identified data set, please explain them in detail (e.g., data contain potentially identifying or sensitive patient information, data are owned by a third-party organization, etc.) and who has imposed them (e.g., a Research Ethics Committee or Institutional Review Board, etc.). Please also provide contact information for a data access committee, ethics committee, or other institutional body to which data requests may be sent.

b) If there are no restrictions, please upload the minimal anonymized data set necessary to replicate your study findings to a stable, public repository and provide us with the relevant URLs, DOIs, or accession numbers. Please see <http://www.bmj.com/content/340/bmj.c181.long> for guidelines on how to de-identify and prepare clinical data for publication. For a list of recommended repositories, please see <https://journals.plos.org/plosone/s/recommended-repositories>. You also have the option of uploading the data as Supporting Information files, but we would recommend depositing data directly to a data repository if possible.

Please update your Data Availability statement in the submission form accordingly.

**Author’s response:** Thank you for this comment, we have added the following section to the data availability statement:

**Data availability statement:**
The data generated in this study consist of observational field notes from observations conducted in birthing rooms and qualitative interview transcripts with healthcare professionals, both of which contain personal data. Data sharing is therefore restricted in accordance with the ethical approval for the study and applicable Swedish regulations governing research data and the protection of personal data. Data may be made available upon reasonable request, subject to the necessary ethical approval and applicable data protection requirements. Enquiries may be directed to Malin Edqvist, Department of Women’s and Children’s Health, Karolinska Institutet

5. Please amend either the title on the online submission form (via Edit Submission) or the title in the manuscript so that they are identical.

**Author’s response:** Thank you for pointing this out. The title is now identical in the manuscript and the online submission form.

6. Your ethics statement should only appear in the Methods section of your manuscript. If your ethics statement is written in any section besides the Methods, please delete it from any other section.

**Author’s response:** Thank you for addressing this issue. The ethics statement has been removed from the Declarations section and now appears only in the Methods section.

7. Please include captions for your Supporting Information files at the end of your manuscript, and update any in-text citations to match accordingly. Please see our Supporting Information guidelines for more information: <http://journals.plos.org/plosone/s/supporting-information>.

**Author’s response:** Captions for all Supporting Information files have now been added at the end of the manuscript, and the corresponding in-text citations have been updated accordingly **(p 21):**

# **Supporting information**

**S1 Table.** Overview of data collection by period and method

**S2 Table.** Overview of the reason for conducting a huddle, number of care providers, stage of labour, and levels of engagement for each huddle in the observations

**S3 Appendix.** Interview guide

8. If the reviewer comments include a recommendation to cite specific previously published works, please review and evaluate these publications to determine whether they are relevant and should be cited. There is no requirement to cite these works unless the editor has indicated otherwise. 
**Author’s response:** Thank you for this clarification. No specific publications were recommended for citation by the reviewers.

9. Please review your reference list to ensure that it is complete and correct. If you have cited papers that have been retracted, please include the rationale for doing so in the manuscript text, or remove these references and replace them with relevant current references. Any changes to the reference list should be mentioned in the rebuttal letter that accompanies your revised manuscript. If you need to cite a retracted article, indicate the article’s retracted status in the References list and also include a citation and full reference for the retraction notice.
**Author’s response:** Thank you for this comment. None of the cited publications have been retracted. The reference list has been carefully reviewed, and the following corrections were made:

**(P. 19, Reference 14):** Marzban S., et al., *Impact of Patient Engagement on Healthcare Quality: A Scoping Review*. J Patient Exp, 2022. **9**: p. 1-12.

**(P. 20, Reference 27):** Takyi E., *The Challenge of Involvement and Detachment in Participant Observation*. The Qualitative Report. 2015;**20**(6), 864-872. Retreived from <http://nsuworks.nova.edu/tqr/vol20/iss6/12>.

**(P. 21, Reference 43):** Kawulich B.B., *Participant observation as a data collection method.* Forum Qualitative Social Research (FQS). 2005;**6**(2), Art. 43.
